# Dectin-1 Compromises Innate Responses and Host Resistance against *Neospora caninum* Infection

**DOI:** 10.3389/fimmu.2017.00245

**Published:** 2017-03-07

**Authors:** Murilo Vieira da Silva, Flávia Batista Ferreira França, Caroline Martins Mota, Arlindo Gomes de Macedo Júnior, Eliézer Lucas Pires Ramos, Fernanda Maria Santiago, José Roberto Mineo, Tiago Wilson Patriarca Mineo

**Affiliations:** ^1^Laboratory of Immunoparasitology “Dr. Mário Endsfeldz Camargo”, Department of Immunology, Institute of Biomedical Sciences, Federal University of Uberlândia, Uberlândia, Brazil

**Keywords:** CLEC7A, IL-12p40, reactive oxygen species, *Neospora caninum*, *Toxoplasma gondii*, macrophages, dendritic cells, laminarin

## Abstract

*Neospora caninum* is an intracellular protozoan parasite that has drawn increasing interest due to its association with worldwide repetitive bovine abortions, which cause billionaire losses to the meat and dairy industries annually. Innate immunity plays an important role in infection control, and *N. caninum* activates the production of inflammatory mediators through toll-like receptors, NOD-like receptors, and mitogen-activated protein kinase signaling pathways. Advances in the knowledge of initial host–parasite interactions are desirable for the design of control measures against the infection, obliterating its pathogenesis. In that sense, we here aimed to describe the role of the innate C-type lectin receptor Dectin-1 during the infection by *N. caninum*. With that intent, we observed that the absence of Dectin-1, observed in genetically depleted (Dectin-1^−/−^) mice or competitively inhibited by an inert agonist [laminarin (LAM)], rescued 50% of the mice infected with lethal doses of *N. caninum*. Dectin-1^−/−^ and LAM-treated mice also presented a reduction in the parasite load during acute and chronic phases, associated with decreased inflammatory scores in the central nervous system. Among all the cell phenotypes that migrated to the initial site of infection, dendritic cells and macrophages gained subpopulations with high Dectin-1 surface expression. The impairment of the receptor in these cells led to a decreased parasite burden, as well as augmented production of IL-12p40. We also found that Dectin-1^+^ cells produced less reactive oxygen species (ROS) at the initial site of the infection, while mice deficient in NADPH oxidase isoform 2 (NOX2^−/−^) were not able to control parasite replication and produce IL-12p40, even upon LAM treatment. Interestingly, the absence of functional Dectin-1 did not alter the susceptibility of mice against closely related *Toxoplasma gondii*. In conclusion, the gathered data suggest that Dectin-1 is involved in the parasite-induced downmodulation of ROS, and other key molecules triggered for the control of *N. caninum* infection and are a promising target for future development of protocols intended for intervention against neosporosis.

## Introduction

Apicomplexa parasites are important pathogens of animals and humans that cause diseases with widespread impact on global health (1). *Neospora caninum* and *Toxoplasma gondii* belong to this phylum. *N. caninum* was described initially as a causative agent of neurological diseases in dogs (2, 3). Currently, this protozoan is recognized worldwide as an important infectious cause of abortions in cows (4), generating significant losses to cattle raising farms aimed at milk and meat production (5), due to its highly efficient vertical transmission (6–8), whereas it seems to be apathogenic to humans (9, 10). *T. gondii* may cause illness in infected humans with a wide variety of clinical symptoms, as a mild, flu-like illness with low-grade fever, myalgia, malaise and headache, ranging to spontaneous abortions, mental and psychomotor retardation of newborns, retino-choroiditis, encephalitis, and hepatitis in adult individuals (11–13). Although phylogenetically related, these parasites show significant biological differences (14), as the reported distinctions in surface carbohydrate content, while *N. caninum* is highly glycosylated, *T. gondii* present low surface carbohydrate content (15).

According to *in vivo* and *in vitro* studies, protective immune responses against the infection by *N. caninum* are typically dependent of a Th1 profile, mediated by the production of proinflammatory cytokines IL-12p40 and IFN-γ, similar to that observed for *T. gondii* and other Apicomplexan parasites (16, 17). During the early stages of the infection, an appropriated innate immune response is mandatory for efficient parasite control, presenting hallmarks as antigenic recognition, presentation, and production of effector molecules as reactive oxygen species (ROS) and nitric oxide (NO). However, pathogens are eventually able to regulate the production of these molecules as an escape mechanism (18–20).

In different infectious processes, innate immune cells are able to directly recognize microbes through the interaction of pathogen-associated molecular patterns and pattern-recognition receptors (PRRs) (21–23). Due to this process, *N. caninum* and *T. gondii* are known to activate different PRRs during the infection, as toll-like receptor 2 (24), TLR3 (25), TLR4 (26), TLR11 (27), TLR12 (28, 29), as well as by cytosolic sensors as nucleotide-binding oligomerization domain containing 2 (30, 31), NOD-like receptor (NLR), NLR family pyrin domain containing 1 (NLRP1), and 3 (NLRP3) inflammasomes (32–34).

Dectin-1 is a C-type lectin, also known as CLEC7A, expressed by myeloid cells (dendritic cells, macrophages, and neutrophils), which specifically recognizes 1,3-β-glucan in a calcium-independent manner (35–37). After interaction between pathogens and Dectin-1, a transmembrane signaling promotes distinct cellular functions, as uptake and killing, production of cytokines, chemokines, and free radicals (38). Dectin-1 is associated with recognition of fungal antigens such as *Candida albicans* and *Aspergillus fumigatus*, as well as other pathogens such as *Mycobacterium tuberculosis*. However, the agonists derived from these pathogens are yet to be described (39–41).

Studies that demonstrate effective participation of this receptor in the immune response against protozoa are scarce in the literature. Evidence of the participation of Dectin-1 in infections by *Leishmania infantum* is still unclear, due to a dubious phenotype (42). Here, we demonstrate that Dectin-1 is related to evasion mechanisms triggered by *N. caninum* against the immune response mounted to protect the hosts, promoting the downregulation of IL-12p40 and effector molecules, consecutively compromising host resistance. On the other hand, Dectin-1 is not required for host’s resistance against closely related *T. gondii*.

## Materials and Methods

### Ethics Statement

All studies involving mice were approved by the animal research ethics committee at UFU (Comitê de Ética na Utilização de Animais da Universidade Federal de Uberlândia—CEUA/UFU), under protocol number 109/16. All procedures including housing and welfare were carried out in accordance with the recommendations in the Guiding Principles for Biomedical Research Involving Animals of the International Council for Laboratory Animal Science (ICLAS), countersigned by the Conselho Nacional de Controle de Experimentação Animal (CONCEA; Brazilian National Consul for the Control of Animal Experimentation) in its E-book (http://www.mct.gov.br/upd_blob/0238/238271.pdf). The UFU animal facility (Centro de Bioterismo e Experimentação Animal—CBEA/UFU) is accredited by the CONCEA (CIAEP: 01.0105.2014) and Comissão Técnica Nacional de Biossegurança (CTNBio, Brazilian National Commission on Biosecurity; CQB: 163/02).

### Animals

For the experiments done in this work, we have used wild-type (WT) mice on a C57BL/6 background, along with genetically deficient littermates in Dectin-1 (Dectin-1^−/−^), and NADPH oxidase isoform 2 (NOX2^−/−^), with 6–8 weeks old. All mice were bred and maintained at CBEA/UFU, in groups of maximum five animals inside each isolator cages, with light/dark cycle of 12 h, food, and water *ad libitum*.

### Parasite Maintenance

*Neospora caninum* tachyzoites (Nc-1 isolate) and *T. gondii* tachyzoites (RH, Pru, and Me49 strains) were maintained by continuous passages in a diploid-immortalized cell line derived from cervical cancer (HeLa; CCL-2, ATCC, Manassas, VA, USA). Briefly, HeLa cells were cultured in RPMI-1640 medium (Thermo Scientific Inc., Waltham MA, USA) supplemented with 25mM HEPES, 2 mM l-glutamine, 100 U/mL penicillin, 100 μg/mL streptomycin, and 10% heat-inactivated calf fetal serum [fetal calf serum (FCS); Thermo Scientific] in an incubator under controlled temperature and atmosphere (37°C, 5% CO_2_, 95% relative humidity; Thermo Scientific). After HeLa infection with *N. caninum*, these cells were cultured in RPMI-1640 medium without FCS or, after infection with different strains of *T. gondii*, with 2% FCS. Extracellular parasites were washed twice (720 × *g*, 10 min, 4°C) with phosphate-buffered saline (PBS, 0.01M, pH 7.2), and the resulting pellet was resuspended in RPMI. Finally, the parasites were suspended in RPMI-1640 medium, and numbers of viable tachyzoites were determined by Trypan blue exclusion (Sigma-Aldrich, St. Louis, MO, USA) in a hemocytometer (43).

In order to obtain tissue cysts of the type II *T. gondii* Me49 strain, we maintained mice chronically infected with sub-lethal doses of the parasite. After 30–45 days of infection, the brains were removed, homogenized, and washed in sterile PBS (pH 7.2) at 1,000 × *g* for 10 min. Tissue cysts were then counted under light microscopy for the experimental infection protocols. For *in vivo* assays involving survival and parasite burden, mice were infected through oral route (gavage) with 20 and 5 cysts, respectively (44, 45).

### Bone Marrow-Derived Macrophages (BMDMs) and Dendritic Cells

Bone marrow-derived macrophages were generated from bone marrow stem cells of WT and Dectin-1^−/−^ mice, as previously described (46). Briefly, stem cells were cultured on 10 cm-diameter polystyrene plates, for 7 days in RPMI-1640 medium, containing HEPES 15 mM, 2 g of sodium bicarbonate/L, 1 mM l-glutamine, supplemented with 20% heat-inactivated FCS and 30% cell-conditioned medium, obtained from the supernatant of confluent L929 cells (LCCM). Differentiated BMDMs were removed from the substrate by vigorous pipetting of ice-cold PBS. Cells were counted and used in the proportion of 1 × 10^6^/mL for the experiments, in 96-well plates.

Bone marrow-derived dendritic cells (BMDCs) were generated as previously cited (24). Briefly, bone marrow stem cells were cultured on 10 cm-diameter polystyrene plates in RPMI-1640 medium supplemented with 10% FCS and murine granulocyte-macrophage colony-stimulating factor (25 ng/mL; BD Biosciences, San Jose, CA, USA). On day 3, supernatant was gently removed and replaced with the same volume of supplemented medium. On day 6, non-adherent cells were removed and placed in 96-well plates (1 × 10^6^ cells/mL) prior to stimulation.

### Dectin-1 Expression

On the third day of infection, peritoneal cells from WT mice infected with 1 × 10^7^ Nc-1 tachyzoites were collected and stained for phenotypic characterization and Dectin-1 expression. Briefly, mice were euthanized, and their peritoneal cavities were washed with ice-cold PBS. The suspension was then centrifuged at 400 × *g*, at 4°C, for 10 min. The cell pellet was resuspended in PBS with 5% of normal rabbit serum, at room temperature, for 15 min, prior to incubation with the appropriate antibodies: anti-CD11b-APC-Cy7, anti-CD11c-FITC, anti-MHCII-PE, anti-CD11c-V450, anti-CD19-APC-Cy7, anti-CD3-Pacific Blue, anti-CD49b-APC (BD Biosciences), and anti-Dectin-1-PE (R&D Systems, Minneapolis, MN, USA). Cells were incubated with primary antibodies conjugated to the different fluorochromes for another 30 min, at room temperature. After washing, cells were suspended in PBS with 3% formaldehyde, read by flow cytometry (FACSCantoII, BD Biosciences), and analyzed using dedicated software (FlowJo X, Tree Star Inc., Ashland, OR, USA).

### ROS and NO Detection

Reactive oxygen species and NO production by peritoneal cells after 3 days of infection were determined through fluorescent probes [CellROX Green Reagent (Thermo Scientific) and DAF-2 DA (Sigma-Aldrich), respectively], according to the manufacturers’ instructions. Briefly, peritoneal cells obtained from infected mice were incubated with each probe for 30 min, at 37°C, followed by four washing cycles with PBS. The stained cell suspension was read by flow cytometry (FACSCantoII, BD Biosciences) and analyzed by dedicated software (FlowJo, Tree Star). Additionally, *in vitro* experiments were also undertaken, in order to determine ROS production in BMDMs generated from WT and Dectin-1^−/−^ mice. Briefly, BMDMs (10^6^ cells/mL) were infected with live Nc-1 tachyzoites (0.5:1, parasite:cell ratio) for 1 h, at 37°C, 5% CO_2_, pretreated or not with laminarin (LAM) (500 μg/mL) for 3 h. After incubation, the supernatant was removed, and the cells were incubated with the probe for 30 min, at 37°C, followed by six washing cycles with PBS. The reaction was read at a plate reader (M2e, Molecular Devices, Sunnyvale, CA, USA) under 488 nm emission and 525 nm excitation.

### IL-12p40 Determination

IL-12p40 production was analyzed in the peritoneal fluids and spleen homogenates of naïve and infected mice, as well as the supernatants harvested from cultured BMDCs and BMDMs. The samples were collected and immediately stored at −70°C until being assayed according to the manufactures’ instructions (Opteia set, BD Biosciences).

### Laminarin Treatment

*In vivo* LAM (Sigma-Aldrich) treatment was performed during seven consecutive days. After the fourth day of treatment, mice were challenged with non-lethal (1 × 10^6^) or lethal (1 × 10^7^) doses of *N. caninum* tachyzoites, according to the aim of each assay. The dose of LAM used was 1 mg/kg/day, by intraperitoneal route. For survival analysis, mice were followed twice a day, until the 30th day of infection, for clinical symptoms compatible with the infection. After advancement in morbidity scores and before the occurrence of natural death induced by the experimental challenge, mice were euthanized to avoid excessive suffering.

### Determination of Parasite Burden

For acute parasitism analysis, we stained the parasites with fluorescent reactive ester dyes, to allow the identification of parasite-associated cells as previously described (43). Briefly, approximately 3 × 10^7^ tachyzoites/mL was pre-stained with CFDA-SE (5 μM/mL; CFSE; Thermo Scientific). After 10 min at 37°C, the tachyzoites were washed with 10 mL of RPMI-1640 with 10% FCS and centrifuged at 800 × *g*, for 10 min, at 4°C. Viable tachyzoites were determined with the Trypan blue exclusion test and used to infect mice, BMDCs and BMDMs. For *in vivo* assays, mice were intraperitonally infected with 1 × 10^7^ CFSE^+^ Nc-1 tachyzoites, and the peritoneal cells were extracted on the third day of infection for analysis of CFSE-associated cells gated within the monocyte region, determined by gating under FSC-A and SSC-A parameters. *In vitro*, BMDMs were infected with CFSE^+^ Nc-1 tachyzoites (MOI 1) and analyzed after 24 h of infection. The parasitism was given as percent or intensity of CFSE^+^ cells after being read in a flow cytometer (FACSCantoII, BD) and analyzed by dedicated software (FlowJo, Tree Star).

Chronic parasitism was determined in brain tissue from mice after 30 days of infection with *N. caninum* or *T. gondii* by real-time quantitative polymerase chain reaction as described elsewhere (47). Primer pairs designed for the Nc-5 region (Np6/Np21) of *N. caninum* (sense: 3′-GCTGAACACCGTATGTCGTAAA-5′; antisense: 3′-AGAGGAATGCCACATAGAAGC-5′) and *T. gondii*’s B1 gene (sense: 3′-GCTCCTCCAGCCGTCTTG-5′; antisense: 3′-TCCTCACCCTCGCCTTCAT) were used in assays based on SYBR green detection system (Promega Co., Madison, WI, USA). DNA extraction was performed from 20 mg of murine brain tissues using a commercial kit (Wizard SV Genomic DNA kit, Promega) according to the manufacturer’s instructions. DNA concentrations were determined by UV spectrophotometry (260 nm; Nanodrop Lite, Thermo Scientific) and adjusted to 200 ng/μL with sterile DNAse free water. Assays to determine *N. caninum* and *T. gondii* parasite loads were performed through real-time PCR (StepOnePlus, Thermo Scientific) and calculated by interpolation from a standard curve with DNA equivalents extracted from tachyzoites included in each run. Brain tissue from naïve mice was analyzed in parallel as negative control.

### Determination of Inflammatory Scores in Brain Tissue

Brain tissue sections obtained from mice after 30 days of infection were stained with hematoxylin and eosin and examined by light microscopy to detect tissue damage. Inflammatory scores were represented as arbitrary units: 0–1, mild; 1–2, moderate; 2–3, severe; and >3, very severe; according to previously described protocol (48). Brain tissue samples from naïve mice served as negative controls.

### Statistical Analysis

Statistical significance of the results was checked using non-parametric one-way analysis of variance followed by Bonferroni’s *post hoc* test (for three or more groups) comparing all pairs of columns, or two-tailed Student’s *t*-test (for two groups). Differences in survival rates between groups were compared using Kaplan–Meier survival analysis, through a log-rank Mantel–Cox test. In all measurements, a *p* value < 0.05 was deemed as statistically significant. Analysis was performed with the aid of commercial software (GraphPad Prism, San Diego, CA, USA).

## Results

### Absence of Dectin-1 Induce Greater Resistance against *N. caninum* Infection in Mice

**Figure 1:**
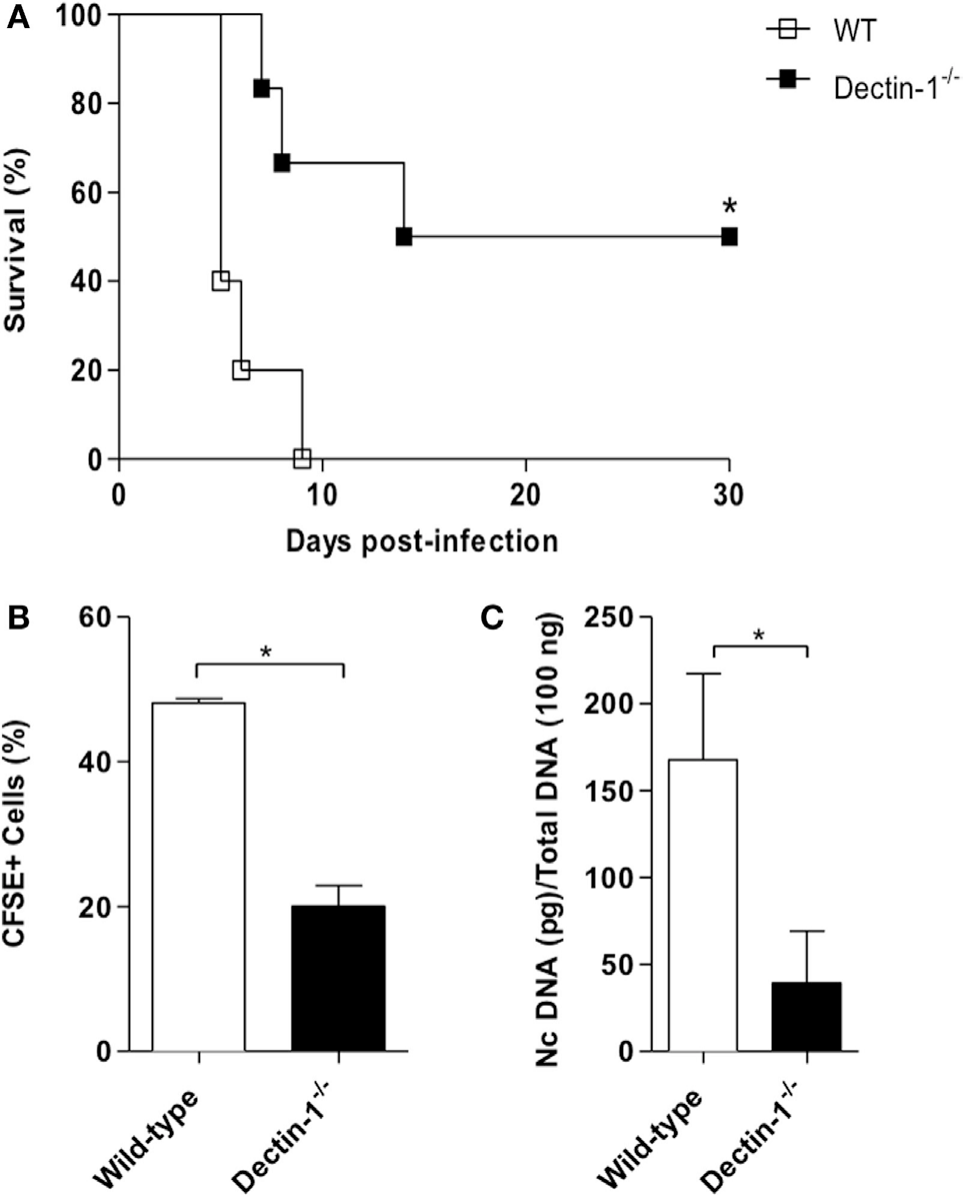
**The absence of Dectin-1 protects mice against *Neospora caninum* infection**. Wild-type (WT) and Dectin-1^−/−^ mice were infected with *N. caninum* (DL100, 1 × 10^7^ tachyzoites/mice) for survival analysis and non-lethal dose (1 × 10^6^ tachyzoites/mice) for acute and chronic parasitism determination. **(A)** Survival curves (10 mice/group). Differences in survival rates between groups were compared using Kaplan–Meier survival analysis, through a long-rank Mantel–Cox test. **(B)** Acute parasitism in peritoneal cells on the third day after infection with CFSE-stained tachyzoites, determined by flow cytometry (6 mice/group). **(C)** Brain parasite load after 30 days of infection (6 mice/group), analyzed by real-time PCR. Results are representative of three independent experiments and expressed as mean ± SEM. *Statistically significant differences (*p* < 0.05).

Dectin-1 is an innate receptor related until now with β-glucan recognition. Its role as a PRR has been described in infections by fungal, bacteria, and some protozoa. With that intent, our initial goal was to evaluate the role of this receptor in mice survival and parasite burden during lethal infection protocol (DL100) with *N. caninum* tachyzoites. Dectin-1^−/−^ mice were more resistant to infection (*p* = 0.0090), since 50% of the genetically deficient mice survived the DL100 challenge (Figure 1A). Our next step was to evaluate if Dectin-1 also interfered with the acute and chronic parasitism, during a non-lethal infection protocol. Dectin-1^−/−^ mice presented a decreased number of parasitized (CFSE^+^) cells in the peritoneal cavity during the acute phase (*p* < 0.01, Figure 1B), and lower concentration of parasite genomic DNA during chronic infections (*p* = 0.0002, Figure 1C), demonstrating that the presence of functional Dectin-1 contributes to the susceptibility of mice to the infection by *N. caninum*.

**Figure 2:**
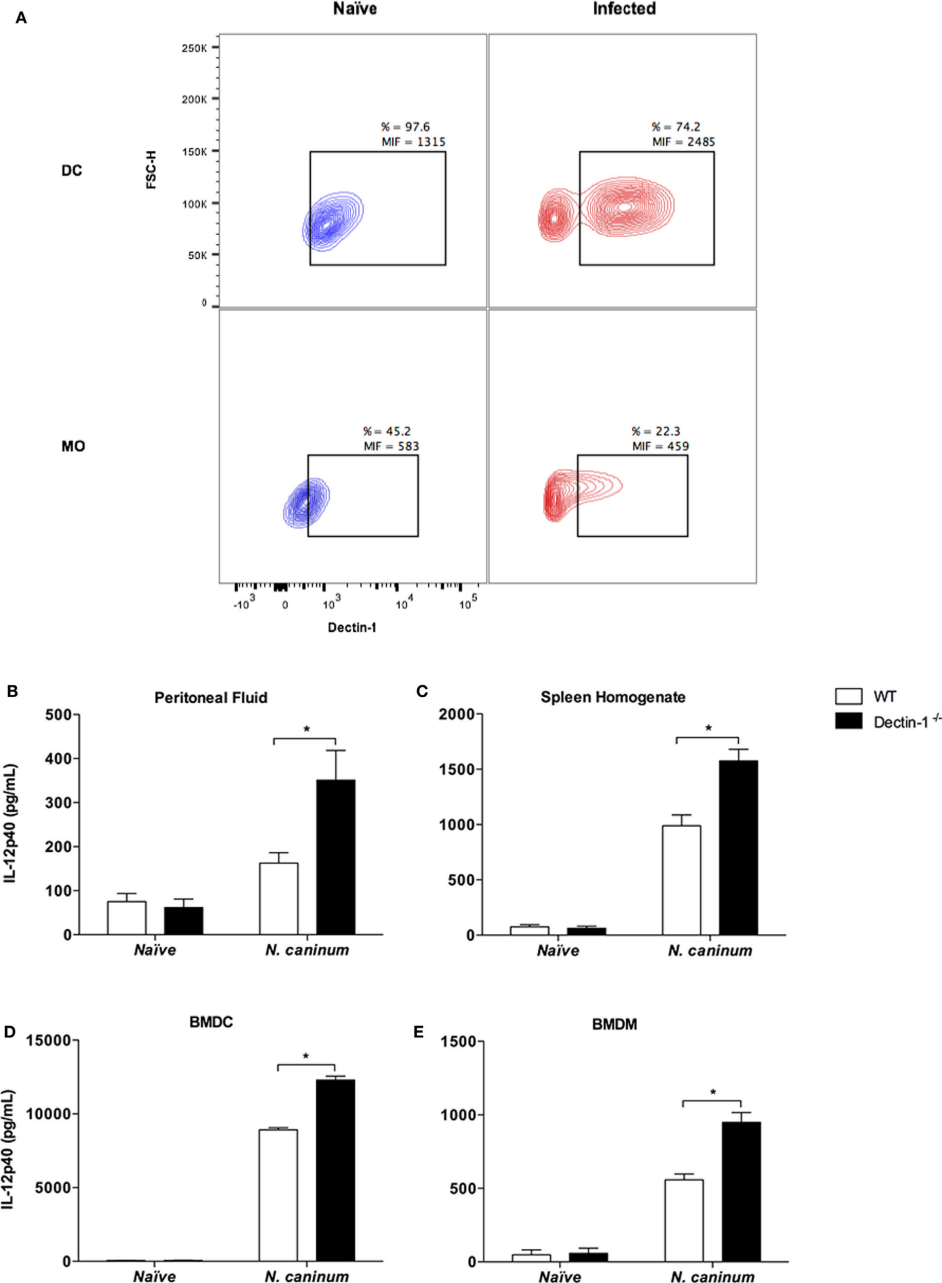
***Neospora caninum* infection alters Dectin-1 expression and IL-12p40 production in dendritic cells and macrophages**. Wild-type (WT) and Dectin-1^−/−^ mice were infected with non-lethal dose of *N. caninum* tachyzoites (1 × 10^6^ tachyzoites/mice) for the analysis of surface Dectin-1 expression in different cellular phenotypes and IL-12p40 determination after 3 days of infection. **(A)** Dectin-1 expression in dendritic cells (CD11b^+^CD11c^+^) and macrophages (CD11b^+^CD11c^−^) collected from the peritoneal cavity of infected WT mice. The results are expressed as the percentage of positive cells (%) and mean intensity of fluorescence (MIF) for Dectin-1, analyzed by flow cytometry. Additionally, IL-12p40 concentration was measured by ELISA in the peritoneal fluid **(B)** and spleen homogenates **(C)** of naïve and infected WT and Dectin-1^−/−^ mice, as well as in the supernatants of bone marrow-derived dendritic cells [BMDCs; **(D)**] and bone marrow-derived macrophages [(BMDMs); **(E)**], after 24 h of incubation with live *N. caninum* tachyzoites (MOI 1). Results expressed as mean ± SEM of three independent experiments. *Statistically significance differences (*p* < 0.05) assessed by analysis of variance followed by Bonferroni multiple comparison posttest.

Since Dectin-1^−/−^ mice presented increased resistance to *N. caninum*, our next step was to evaluate if the expression of the receptor was altered during the acute phase of the infection. For that purpose, WT mice were infected with non-lethal doses of *N. caninum* tachyzoites and evaluated for Dectin-1 expression in different phenotypes of peritoneal cells after 3 days of infection. The most pronounced differences in Dectin-1 expression were observed in antigen-presenting cells (dendritic cells and macrophages), which are the primary inducers of appropriated adaptive immune responses. Resident dendritic cells (CD11b^+^CD11c^+^ cells, gated in the monocyte region) in the peritoneal cavity of naïve mice showed constitutive expression of Dectin-1 in its surface (Dectin-1^+^ DCs = 97.6%). However, during acute infection, two distinct populations were clearly observed (CD11b^+^CD11c^+^Dectin-1^−^ and CD11b^+^CD11c^+^Dectin-1^+^), where the expression of Dectin-1 in positive cells was considerably increased (Figure 2A). The same pattern of events was observed for macrophages (CD11b^+^CD11c^−^ cells, gated in the monocyte region). While a homogenous constitutive expression of the receptor was observed in resident cells obtained from naïve mice, macrophages obtained during acute infection showed polarized cell phenotypes: an increased proportion of Dectin-1^−^ macrophages versus Dectin-1^+^ cells with high expression of the receptor (Figure 2A). No relevant differences in Dectin-1 expression were observed in polymorphonuclear cells and lymphocytes (NK, T, and B; data not shown).

Given the differences observed in Dectin-1 expression induced by the infection in antigen-presenting cells, our next question was if the presence of the functional receptor altered IL-12p40 production, an important proinflammatory mediator related to the control of parasite replication during the acute phase of the infection. With that intent, WT and Dectin-1^−/−^ mice were infected with non-lethal doses of *N. caninum* tachyzoites for the assessment of IL-12p40 production after 3 days of infection. Our results showed increased levels of this cytokines in the peritoneal fluid (*p* < 0.001, Figure 2B) and spleen homogenates (*p* < 0.001, Figure 2C), when compared to WT mice.

Due to the previous observations, and taken that antigen-presenting cells are crucial for the induction of proper Th1-biased immune responses against *N. caninum*, we then aimed to evaluate whether Dectin-1 would directly affect the ability of dendritic cells and macrophages to increase the production of IL-12p40, a crucial molecule during antigen presentation in *N. caninum* infection. For that, we undertook experiments, which exposed BMDCs and BMDMs, obtained from WT and Dectin-1^−/−^ mice, to live *N. caninum* tachyzoites *in vitro*. In that context, we observed that Dectin-1^−/−^ BMDC (Figure 2D) and BMDM (Figure 2E) presented augmented production of IL-12p40 (*p* < 0.001), compared to WT cells after incubation with *N. caninum* live tachyzoites for 24 h.

Taken together, these data suggest that *N. caninum* tampers with Dectin-1 expression in antigen-presenting cells, negatively regulating the production of crucial molecules for the mounting of effective immune responses against the infection.

### Laminarin Is Effective in the Control of *N. caninum* Infection in an ROS-Dependent Manner

**Figure 3:**
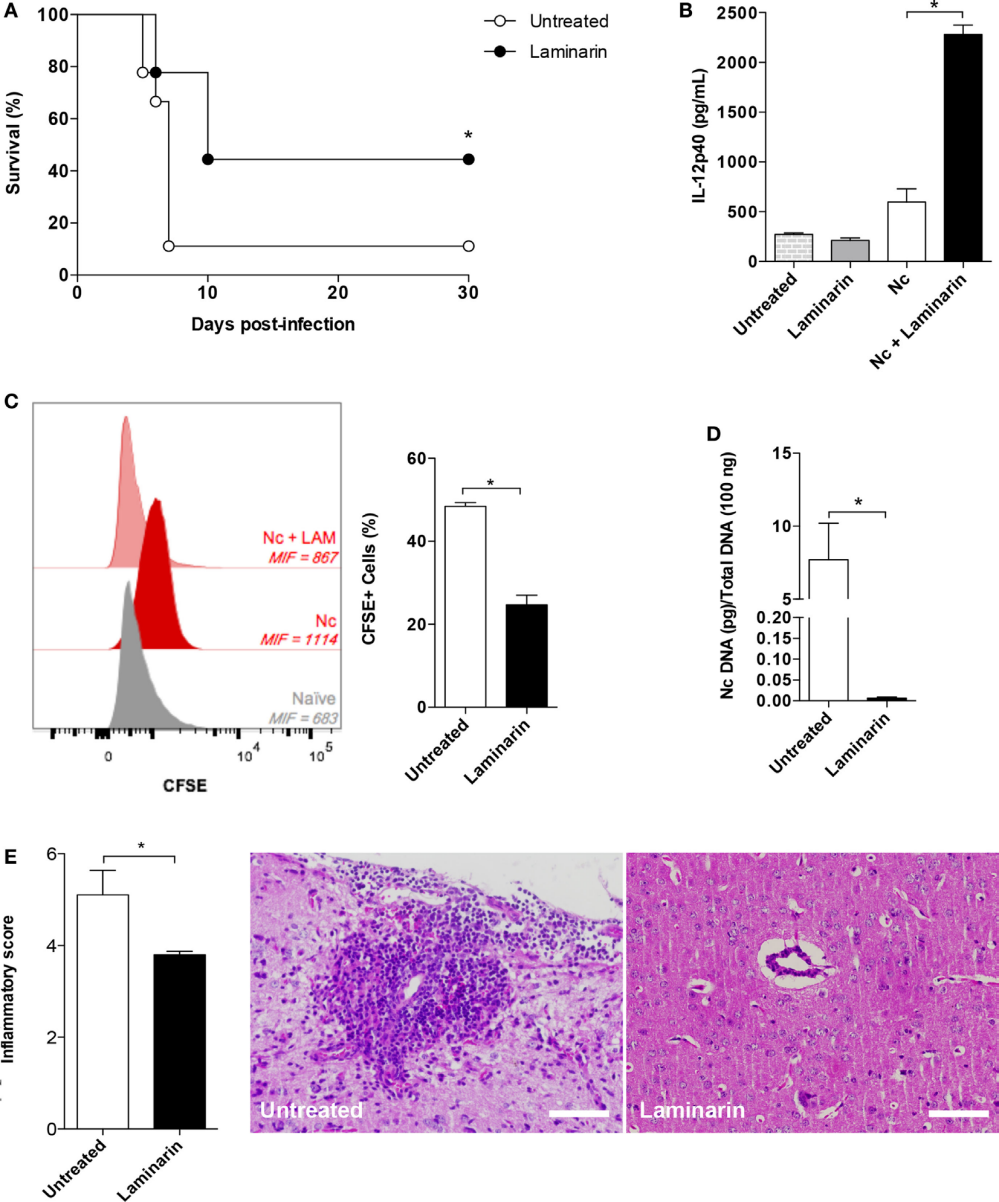
**Laminarin (LAM) is effective in the control of *Neospora caninum* infection**. Wild-type mice were treated or not with LAM (1 mg/kg/day) during seven consecutive days. In the fourth day of treatment, mice (treated or not) were infected (10 mice/group) with 1 × 10^7^ *N. caninum* tachyzoites for survival analysis **(A)**. Differences in survival rates between groups were compared using Kaplan–Meier, through a long-rank Mantel–Cox test. **(B)** IL-12p40 concentration was determined by ELISA in the peritoneal fluid of mice, at the third day of a non-lethal infection (1 × 10^6^ tachyzoites/mice). Results were expressed as mean ± SEM and are representative of three independent experiments. *Statistically significance differences (*p* < 0.05), assessed by analysis of variance followed by Bonferroni multiple comparison posttest. **(C)** Acute parasite burden determined by the mean intensity of fluorescence (MIF) of peritoneal cells after 3 days of a non-lethal infection (1 × 10^6^ tachyzoites/mice) with CFSE-stained tachyzoites, determined by flow cytometry (6 mice/group). **(D)** Parasite load in the central nervous system of mice after 30 days of infection (6 mice/group), analyzed by real-time quantitative polymerase chain reaction. **(E)** Mean inflammatory scores attributed to the different groups after 30 days of infection, along with representative photomicrographs of lesions found. Results are representative of three independent experiments. *Statistically significant differences (*p* < 0.05).

Once we observed that the absence of Dectin-1 is associated with increased resistance of the host against *N. caninum* infection, due to an increment of IL-12p40 linked to the infection control, we next aimed to assess whether the use of LAM, a competitive inhibitor of Dectin-1, would be suitable to be employed in therapeutic measures against the parasite. Treatment with LAM rescued 50% of the challenged animals with DL100 (*p* = 0.0415), similar to the result obtained using Dectin-1^−/−^ mice (Figure 3A). In addition, after 3 days of infection, mice challenged with *N. caninum* and treated with LAM presented increased levels of IL-12p40 in the peritoneal fluid (*p* = 0.0159, Figure 3B) associated with decreased parasitism in peritoneal monocytic cells (*p* = 0.0110, Figure 3C) during the acute phase of a non-lethal infection protocol. During the chronic phase of the infection, this phenotype of increased resistance was maintained in mice treated with LAM, which presented a reduced concentration of parasite genomic DNA in the central nervous system (*p* = 0.0097, Figure 3D), associated with a decreased inflammatory score (*p* = 0.0095, Figure 3E).

**Figure 4:**
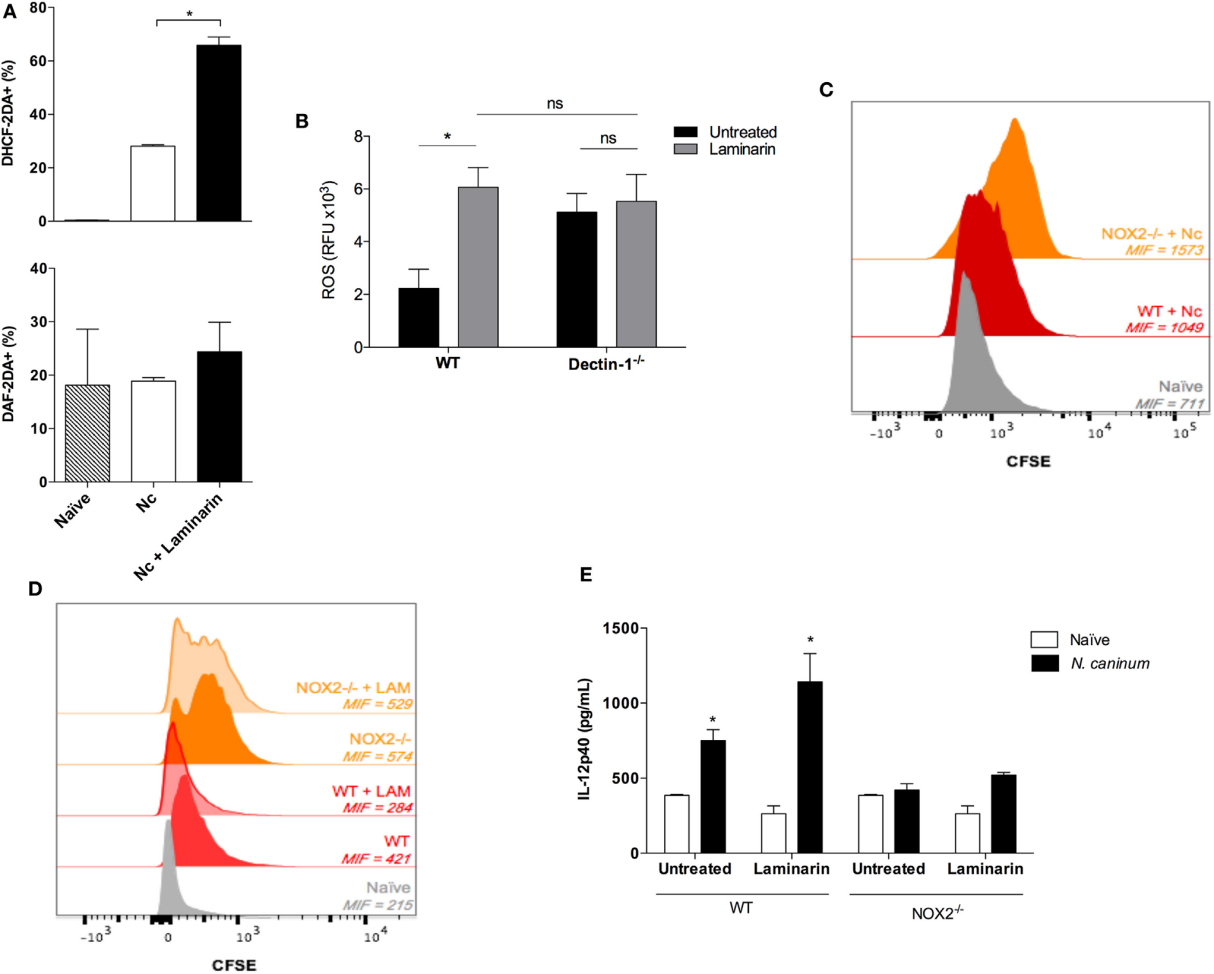
**The protective effect of laminarin (LAM) against *Neospora caninum* infection is dependent on reactive oxygen species (ROS)**. For *in vivo* experiments, wild-type (WT) and/or NADPH oxidase isoform 2 (NOX2)^−/−^ mice were treated or not with LAM (1 mg/kg/day), during seven consecutive days. In the fourth day of treatment, mice were infected with non-lethal doses (1 × 10^6^) of *N. caninum* tachyzoites. For the *in vitro* assay, bone marrow-derived macrophages (BMDMs) (10^6^ cells/mL) were infected with live Nc-1 tachyzoites (0.5:1, parasite:cell ratio) for 1 h, pretreated or not with LAM (500 μg/mL) for 3 h. **(A)** Percentage of ROS and NO positive cells obtained from the peritoneal cavity of WT mice, determined by flow cytometry. **(B)** Liquid production of ROS (RFU reading of infected cells subtracted of each individual control) of BMDMs generated from WT and Dectin-1^−/−^ mice, treated or not with LAM. **(C)** Peritoneal acute phase parasitism in WT and NOX2^−/−^ mice after 3 days of infection with 1 × 10^6^ *N. caninum* tachyzoites stained with CFSE, analyzed by flow cytometry, and expressed as mean intensity of fluorescence (MIF). **(D)** In a similar experiment, WT and NOX2^−/−^ mice were treated or not with LAM, and the acute phase parasite burden was again assessed through MIF of CFSE^+^ cells. **(E)** IL-12p40 concentration was also determined in the peritoneal fluid of WT and NOX2^−/−^ mice, under the same infection and treatment protocol. Results were expressed as mean ± SEM and are representative of three independent experiments. *Statistically significance differences (*p* < 0.05), assessed by analysis of variance followed by Bonferroni multiple comparison posttest.

Based on these findings, we next sought to shed some light into the molecular mechanism rendering protection against *N. caninum* infection, upon blockage of Dectin-1. During infections, it is well described that different effector molecules are related to the elimination of intracellular pathogens, with ROS and NO as hallmarks of this process (38, 49–52). Thus, we evaluated if LAM treatment interfered with the production of these free radicals during *in vivo* experiments with non-lethal infections with live tachyzoites. We observed that LAM significantly increased ROS expression in monocytic peritoneal cells (*p* = 0.0273) but maintained stable percentage of NO^+^ cells after 3 days of infection (Figure 4A). We further confirmed this phenomenon with an *in vitro* experiment (Figure 4B), where we demonstrate that the treatment of WT BMDMs with LAM does significantly increase ROS production (*p* = 0.008) during infection with live tachyzoites, similar to the levels produced by Dectin-1^−/−^ BMDMs. On the other hand, and as expected, LAM did not produce an additive effect in ROS produced by Dectin-1^−/−^ BMDMs (*p* = 0.751). To evaluate if ROS, which has not yet been described during *N. caninum* infection, is related to parasite control, we used WT and genetically deficient mice in NOX2^−^*^/^*^−^ to evaluate their ability to control acute parasite replication using CFSE-stained parasites. As expected, we observed that NOX2^−^*^/^*^−^ mice failed to control parasite replication due to the increased expression of CFSE detected in the peritoneal cells gated in the monocyte region (Figure 4C), demonstrating that ROS is critical for the control of *N. caninum* replication.

To elucidate whether LAM would be able to control parasite replication in a ROS-independent manner, which would indicate that the molecular mechanism behind protection upon Dectin-1 blockage would be performed by distinct molecules, WT and NOX2^−/−^ mice were treated with LAM and infected to evaluate the monocytic peritoneal parasitism with CFSE^+^ tachyzoites. Interestingly, while LAM-treated WT mice presented a decreased peritoneal monocytic parasite burden; the intensity of CFSE^+^ cells in the peritoneal cavity of NOX2^−/−^ remained practically unaltered upon treatment (Figure 4D). Finally, we aimed to correlate if Dectin-1 mediated inhibition of ROS production during *N. caninum* infection would also affect IL-12p40 production in the infected mice. For that, WT and NOX2^−/−^ mice were treated with LAM and infected for 3 days for cytokine measurement in the peritoneal fluid. As already demonstrated, WT mice produced IL-12p40 upon infection, which was significantly increased in LAM-treated mice (*p* = 0.0188). Surprisingly, NOX2^−/−^ mice were not able to respond to the infectious stimulus with the production of the referred cytokine, which was not even partially reverted by LAM (Figure 4E).

In that context, our results suggest that LAM treatment is efficient to control the pathogenesis of the infection in the proposed models, through a mechanism regulated by the increase in ROS production and consequent upregulation of IL-12p40 during the acute phase of the infection.

### *T. gondii* Does Not Promote Host Susceptibility Mediated by Dectin-1

Although closely related, *N. caninum* and *T. gondii* present marked biological distinctions, as its surface carbohydrate contents (15). We then aimed to answer whether the absence of Dectin-1 would protect mice against *T. gondii* infection, in the same fashion as seen for *N. caninum*. For this, a set of *in vivo* experiments using different clonal *T. gondii* strains, doses and routes of infection were carried out in WT and Dectin-1^−/−^ mice. Overall, no differences were noted in susceptibility/resistance of the animals submitted to the infectious protocols.

**Figure 5:**
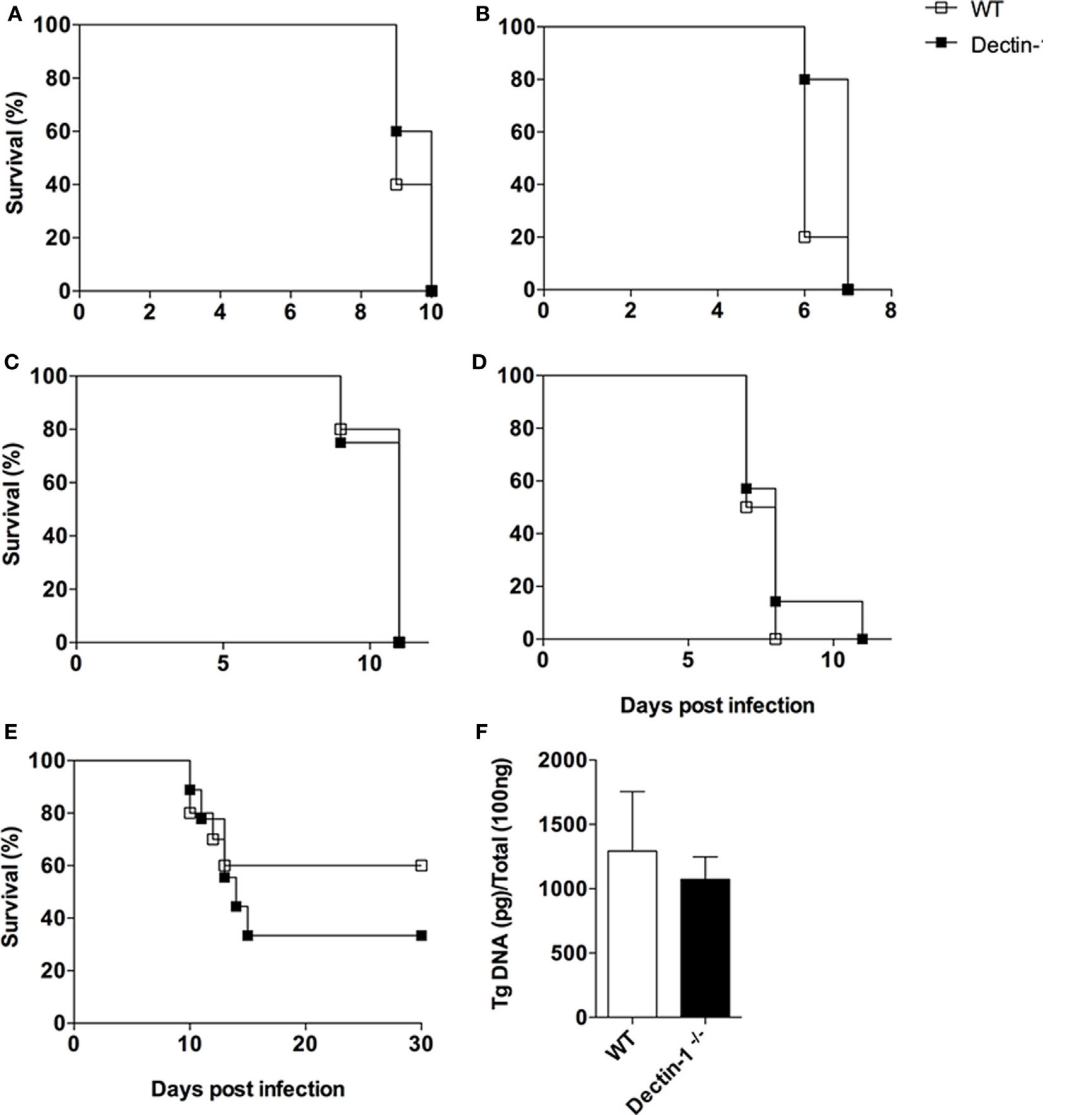
***Toxoplasma gondii* does not promote host susceptibility mediated by Dectin-1**. Wild-type (WT) and Dectin-1^−/−^ mice were infected with different strains, doses, and routes by *T. gondii*, in order to check for differences in survival analysis and parasite burden. Survival curves of **(A)** mice infected by intraperitoneal route with 1 × 10^2^ tachyzoites of RH strain (10 mice/group); **(B)** mice infected by intraperitoneal route with 1 × 10^4^ tachyzoites of Pru strain (10 mice/group); **(C)** mice infected by intraperitoneal route with 1 × 10^3^ and **(D)** 1 × 10^5^ tachyzoites of Me49 strain (10 mice/group); **(E)** mice infected by oral route with 30 cysts of Me49 strain (10 mice/group). **(F)** Chronic phase parasite load of the central nervous system of mice after 30 days of infection with five cysts of Me49 strain (6 mice/group), analyzed by real-time PCR. Survival rates were compared using Kaplan–Meier survival analysis, through a long-rank Mantel–Cox test, but no statistically significance differences were observed. Results for quantitative polymerase chain reaction (qPCR) were expressed as mean ± SEM and are representative of three independent experiments. Statistical significance of the qPCR results was assessed by Student’s *t*-test.

Initially, survival assays were undertaken. WT and Dectin-1^−/−^ mice infected by intraperitoneal route with 1 × 10^2^ tachyzoites of RH strain (type I) succumbed, in a similar fashion, to the challenge, with 100% lethality of the different groups until the 10th day postinfection (Figure 5A). Dectin-1^−/−^ mice were also checked for increased survival against type II strains. WT and Dectin-1 mice were challenged with Pru strain tachyzoites (1 × 10^4^), by intraperitoneal route, with full lethality being observed in both groups after 7 days of infection (Figure 5B).

We also evaluated if the infection protocol was affecting the experimental observations. For that, we adopted Me49 strain (type II) to test distinct infectious doses and routes. WT and Dectin-1^−/−^ mice were then infected with 1 × 10^3^ or 1 × 10^5^ tachyzoites, by intraperitoneal route, and both groups showed similar survival curves, with all animals succumbing to the infection until the 12th day (Figures 5C,D). To evaluate whether the route of the infection would induce differential resistance mediated by Dectin-1, WT and Dectin-1^−/−^ mice were infected by the oral route, with 30 cysts of Me49 strain for survival analysis, or 5 cysts for the evaluation of chronic parasitism in the brain. Once more, our results did not point out differences in survival rates among the groups (Figure 5E), nor in chronic phase parasitism (Figure 5F).

Thus, unlike of the observed for *N. caninum*, these sets of *in vivo* experiments with *T. gondii* demonstrate that Dectin-1 is differentially regulated in infections caused by the two closely related parasites.

## Discussion

During infections by protozoans, the activation of innate immune responses is critical for the development of effective immune response, which culminates in the restriction of microbial multiplication (25). *N. caninum* is an important veterinary pathogen that induces reproductive loses in cattle, as well as neurologic diseases in dogs (5). Given the importance of this parasite, our group has been dedicated in the last years to understand the mechanisms related to immunity against this parasite, especially regarding innate sensors. We have previously demonstrated that toll-like receptors (TLRs) are essential for host resistance against neosporosis, since MyD88 and TLR2 ablation produced pronounced effects over the development of a specific Th1 response, based on the classical IL-12/IFN-γ axis, during the course of infection (24, 53); other classes of receptors—as Nod2—are also relevant in the pathogenesis of the disease (30).

Dectin-1 is a well-characterized β-glucan receptor specially related to fungal recognition. Nevertheless, increasing number of reports suggests that it also has relevant functions during infection by other pathogenic agents, such as bacteria and protozoan. In this work, our major aim was to show that Dectin-1 plays an important role in downmodulating the immune response against *N. caninum*, but not for the phylogenetic-related parasite *T. gondii*. We show that the absence of Dectin-1 rescues mice challenged with lethal doses of *N. caninum* tachyzoites, but no differences were observed in the survival rate of mice infected with distinct stains, routes, or doses of *T. gondii*.

In fact, the role of each PRR is strictly related to the pathogen. This phenomenon has been evidenced in studies with fungi, where simple modifications in antigenic complexes between different strains of *C. albicans* induce significant changes in survival rates in Dectin-1^−/−^ mice (54). To our knowledge, there are no known agonist of Dectin-1 described in *N. caninum* and *T. gondii*. However, the differences in survival and parasite burden obtained for both parasites in our experimental model may be related to the antigenic composition of each parasite, more specifically, the glycosylation and carbohydrate content of surface proteins (15). In order for the host to have greater resistance during the infectious processes, the control of parasite replication is crucial (55). Thus, we associated the higher survival rates observed in Dectin-1^−/−^ mice with their larger ability to control *N. caninum* replication in both acute and chronic stages of infection, fact not observed in *T. gondii* infection.

Dectin-1 is predominantly expressed on the surface of myeloid cells as monocyte/macrophages and neutrophil lineages (56). Here, we observed Dectin-1 expression in different cell phenotypes, at the initial site of infection during the acute phase. As expected, no differences in Dectin-1 expression were observed in lymphoid cells, but *N. caninum* infection induced changes in Dectin-1 expression in populations of dendritic cells and macrophages. We found a decrease in the percentage of cells expressing Dectin-1 after the infection, which changed from homogeneous positive populations to a dichotomy of receptor negative cells and ones with high expression of the PRR. In association, WT (Dectin-1^+^) mice presented dendritic cells and macrophages with reduced IL-12p40 production after contact with live tachyzoites, which by its turn is followed by an increased parasite burden in both acute and chronic phases of infection. An effective control of infections by intracellular pathogens is closely linked to an innate immune response that culminates in the activation of an adaptive response, capable of neutralizing forms of the parasite disseminated through the blood stream and lymphatic system (57, 58). With that intent, antigen presentation assumes a central role in the activation of effective adaptive immune responses (59). Altogether, these data demonstrate that there is a direct or indirect interaction between *N. caninum* and Dectin-1, which compromises the effectiveness of the immune response triggered against the parasite.

Using this rationale, Dectin-1 blockade would be desirable as a potential therapeutic strategy against neosporosis. In this scenario, we suggest that LAM may be tested in the treatment against this infection, once it interacts directly with Dectin-1, is inert, is efficient as we show here and, among the β-1,3-glucans, the compound obtained from *Laminaria digitata* has high solubility in water and presents low polydispersity, characteristics that makes this compound desirable to the pharmaceutical industry (60, 61).

Reactive oxygen species are generated through the activation of diverse signaling pathways, including Dectin-1 and TLR-induced innate immune responses, promoting antimicrobial activity and local inflammation (38, 62–64). It has already been demonstrated that NADPH oxidase-derived ROS plays an essential role in inflammatory responses and anti-parasitic activity against *T. gondii* (62, 64). Additionally, excreted-secreted antigens from *T. gondii* or cell membrane stress due to the parasites’ active invasion mechanism and lytic cycle are efficient in ROS induction (50, 52). However, ROS formation and its consequences in response to *N. caninum* infection had yet to be determined. For the first time, we present here that ROS is important in the control of *N. caninum* replication, since the parasite load in NOX2^−/−^ mice was uncontrolled. We have also shown that Dectin-1 is a negative regulator of ROS during the infection, since competitive inhibition of the receptor specifically increased ROS production by peritoneal cells, while it did not interfere with NO synthesis (65, 66). Furthermore, we show that Dectin-1 downmodulation of ROS hampers its ability to act as a positive regulator of Th1 immune responses, favoring IL-12p40 production and consequent proinflammatory molecules required for parasite restriction (24, 67).

Altogether, the data herein gathered demonstrate that *N. caninum* evades effector immune responses through Dectin-1-mediated dampening of ROS and, consequently, Th1 pathways. We also show that an inert receptor agonist may be used to control the pathogenesis of the infection in mice, which should be transposed for infection models using dogs and cattle—species directly affected by *N. caninum*—in an attempt to reduce abortions and deaths caused by neosporosis.

## Author Contributions

Conceived and designed the experiments: MS, FS, JM, and TM; performed the experiments: MS, FF, CM, FS, AJ, and ER; analyzed the data and wrote the paper: MS, JM, and TM; supplied reagents: JM and TM.

## Conflict of Interest Statement

The authors declare that the research was conducted in the absence of any commercial or financial relationships that could be construed as a potential conflict of interest.
